# Dolichoectatic arterial compression of the chiasm and optic nerve: a case report

**DOI:** 10.3205/oc000223

**Published:** 2023-09-19

**Authors:** Sara Ceuppens, Ine Lambert, Marcel Ten Tusscher

**Affiliations:** 1 Department of Ophthalmology, Universitair Ziekenhuis Brussel, Vrije Universiteit Brussel, Brussels, Belgium

**Keywords:** compressive opticoneuropathy, congenital cerebrovascular anomalies, anterior visual pathway

## Abstract

Dolichoectatic arterial compression of the anterior visual pathway is a rare but recognized condition. The functional impact, however, is still less clear. We describe a case of a 56-year-old male with diminished visual acuity in the left eye and incongruent inferior visual defect in both eyes caused by dolichoectasia due to a congenital hypoplasia of one horizontal segment of the circle of Willis and subsequent arterial compression of the chiasm and left optic nerve.

## Introduction

The term dolichoectasia was introduced to describe a rare condition of the large intracranial vessels at the base of the brain which causes the vessels to be elongated and dilated [1]. 

Compressive optic neuropathies caused by mass lesions like tumors or aneurysms are well-known. Functional vision loss as a result of compression of the intracranial arteries, however, is rare and less well-understood [2], [3]. Compression of the anterior visual pathway (AVP) by intracranial arteries has been observed and reported in radiological studies; the incidence of functional loss as a result remains controversial [2], [3], [4].

To the best of our knowledge, this is the first case describing a dolichoectatic compression of the chiasm and optic nerve likely due to hypoplasia of one A1 segment in the circle of Willis and a congenital asymmetry between the right and left internal carotid. 

## Case description

**Figure 1:**
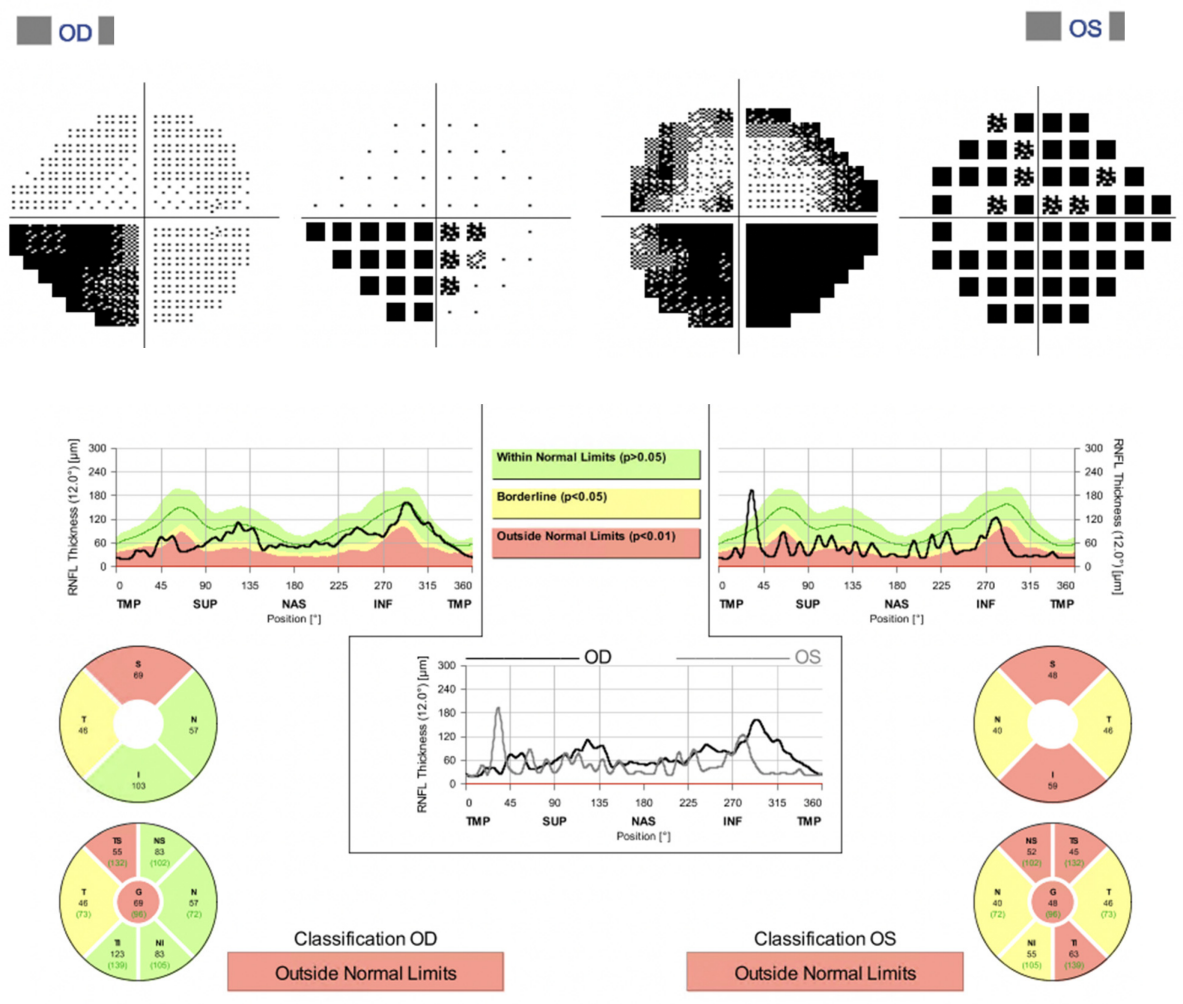
Bilateral incongruent inferior visual field defect and superior arcuate scotoma in the left eye. On OCT thinning of the retinal nerve fiber layer in superotemporal segment of the right eye and more pronounced thinning of the superior segment compared to inferior in the left eye.

A 56-year-old male presented to our clinic with decreased vision in the left eye for several months. Twenty-five years ago he endured an accidental chemical burn of one eye but could not recall which one. He is treated by a rheumatologist for chronic back pain problems. On examination the best corrected visual acuity (BCVA) in the right eye was 10/10 and the left eye 4/10. He had a left relative afferent pupillary defect (RAPD) with an impaired Ishihara color test and a pale optic disc in the left eye. The intra-ocular pressure was 16 mmHg and 15 mmHg and the cup-to-disc ratio (c/d) 0.5 and 0.6 in his right eye and left eye, respectively. The spectral-domain optical coherence tomography (SD-OCT) of the optic nerve head showed a thinning of the retinal nerve fiber layer (RNFL) superotemporally in the right eye, while in the left eye the superior thinning was more pronounced than inferior. His visual field revealed an inferonasal defect in the right eye and an inferior scotoma with a superior arcuate defect in the left eye (Figure 1 (Fig. 1)). 

On the pattern reversal visual evoked potential (pVEP) a decrease in amplitude was seen in the left eye (15’1.4 µV; 60’1.8 µV); the peak times in both eyes and the amplitude of the right eye were within age-corrected normal limits. 

Because of the asymmetry of the optic neuropathy and possible compression of the left optic nerve and chiasm, a magnetic resonance imaging (MRI) was requested.

**Figure 2:**
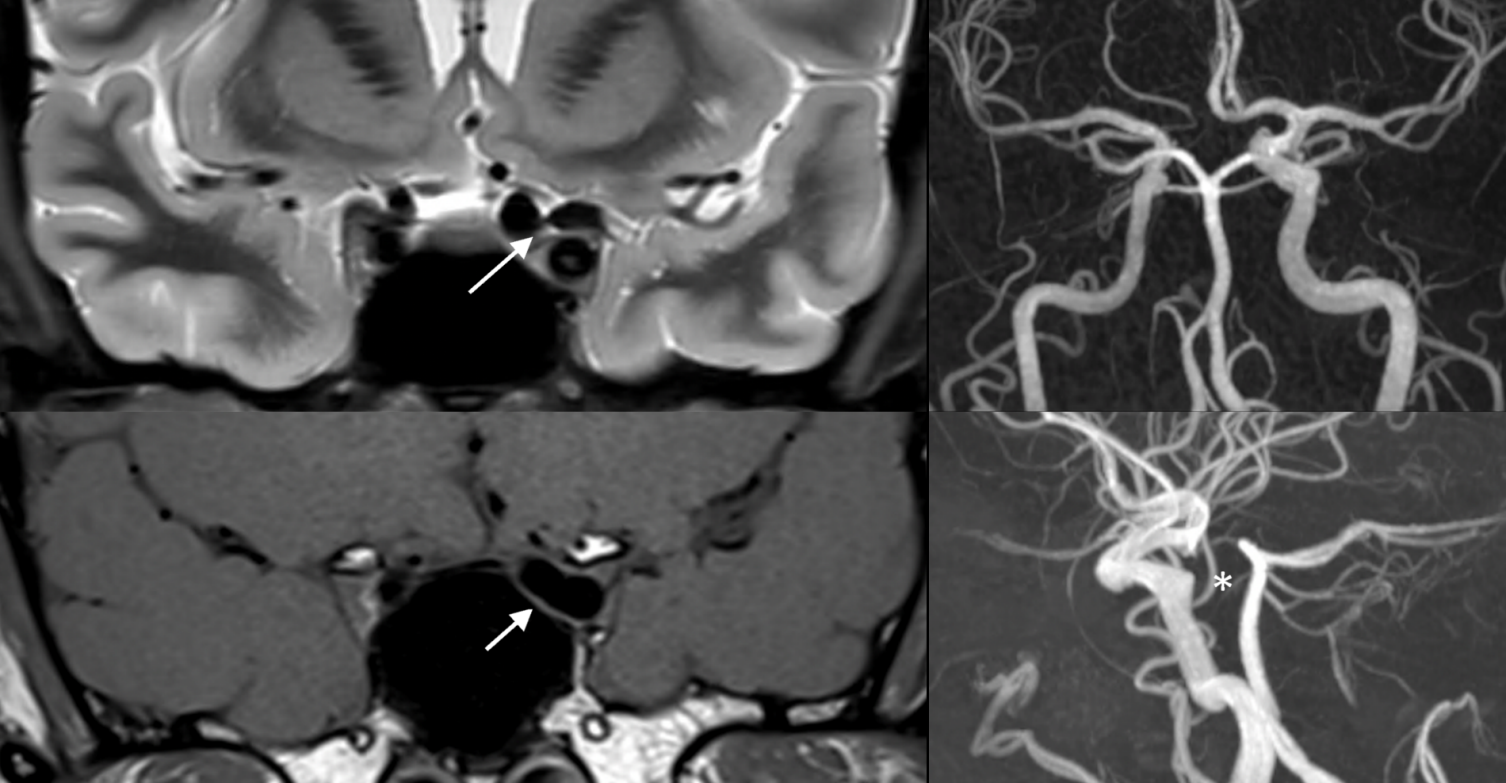
On the left side both arrows show compression of the chiasm and left optic nerve by the dolichoectatic artery in the T2-weighted (above) and T1-weighted (below) MRI in coronal plane. On the right side the angiogram of the asymmetric internal carotid (above) giving rise to an ectatic and elongated left supraclinoid internal carotid artery and the absence of the right A1 segment (below, marked by *).

The MRI and MRI-angio (MRA) revealed a compression of the left optic nerve and chiasm by a dolichoectatic left supraclinoid internal carotid artery (Figure 2 (Fig. 2)). 

On the left side of the chiasm and distal optic nerve a dilated A1 segment of the left anterior cerebral artery and dilated anterior communicating artery with an absence of the right A1 segment were shown. 

This likely congential asymmetric internal carotid artery gives rise to an ectatic and elongated left supraclinoid internal carotid artery, which compresses the chiasm and left optic nerve on the other side. 

After debating surgical options, the patient preferred frequent close evaluations of the visual function while postponing intervention. 

After three months the visual field and OCT of the nerve head appeared stable. A regular three-month follow-up was proposed.

## Discussion

The first case of functional vision loss due to an ectatic intracranial artery was published in 1932 by Caramazza [5]. Since then, three retrospective case studies have described visual loss associated with vascular compression of the AVP [2], [3], [6]. Patients described were of a wide age range (28–91 years old) with a median age at diagnosis of 72 years. Men and women seemed to be equally affected (31 men and 34 women). 

A series of 18 patients with a symptomatic compression of the optic nerve by the internal carotid artery was described by Jacobsen et al. [2]. Regrettably, only two thirds of the patients had undergone visual field testing. In 2004 Purvin et al. described 10 patients with dolichoectatic arterial compression of the AVP, diagnosed with MRI and MRA [3]. Most cases showed compression by the internal carotid artery and less by the basilar artery (3 out of 10 patients). More recently a cohort of 37 patients was described by Jain et al., containing instances of compression by internal carotid artery, anterior cerebral artery, posterior communicating artery, and ophthalmic artery [6]. 

All three case studies conclude a mostly slow progression rate of the visual loss, except for two cases [3].

The mechanism by which nerves are damaged in these cases remains debatable. Direct damage from the vessels’ pulsatility on the nerve fibers or ischaemic damage caused by compression of the local blood supply to the nerve fibers have been suggested [7]. 

Because of the slow progression rate, conservative follow-up with visual field testing is advised. However, vision improved after microvascular decompression in some cases [8], [9].

In the present case a congenital absent right A1 segment with asymmetry of the internal carotid artery resulted in a dolichoectatic left supraclinoid and dilated anterior communicating artery because of the absence of the right A1 segment. These dilated vessels compress the distal part of the left optic nerve and chiasm, causing a loss of visual acuity in the left eye, presence of a RAPD, impaired color testing, and a pale optic disc. The visual field of both eyes is affected with an inferonasal defect and inferior scotoma in the right and left eye, respectively. Repeated visual field testing after 3 months showed no progression.

Vascular compression of the AVP may at presentation mimic normal tension glaucoma (NTG) with paracentral scotomas on visual field testing, showing that a complete and comprehensive neuro-ophthalmological examination with color testing, relative afferent pupillary defect, visual acuity, and evaluation of the optic nerve head is important in all patients with optic neuropathy. Any finding atypical for NTG should warrant further investigation with imaging [10], [11]. 

## Conclusion

Visual field defects and optic nerve changes may be caused by an arterial compression of the anterior visual pathway. Dolichoectatic arterial compression of the optic nerve and chiasm is rare and potentially under-recognized. 

A close follow-up of the patient and interdisciplinary consultation with neurosurgery if progression is detected are key in managing the patient.

## Notes

### Competing interests

The authors declare that they have no competing interests.
